# A cautionary tale: the non-causal association between type 2 diabetes risk SNP, rs7756992, and levels of non-coding RNA, *CDKAL1-v1*

**DOI:** 10.1007/s00125-015-3508-9

**Published:** 2015-01-30

**Authors:** Jonathan M. Locke, Fan-Yan Wei, Kazuhito Tomizawa, Michael N. Weedon, Lorna W. Harries

**Affiliations:** 1 Institute of Biomedical and Clinical Science, University of Exeter Medical School, RILD Building, Barrack Road, Exeter, EX2 5DW UK; 2 Department of Molecular Physiology, Faculty of Life Sciences, Kumamoto University, Kumamoto, Japan

**Keywords:** *CDKAL1*, Expression quantitative trait loci, Genome-wide association studies, Non-coding RNA, Type 2 diabetes

## Abstract

**Aims/hypothesis:**

Intronic single nucleotide polymorphisms (SNPs) in the *CDKAL1* gene are associated with risk of developing type 2 diabetes. A strong correlation between risk alleles and lower levels of the non-coding RNA, *CDKAL1-v1*, has recently been reported in whole blood extracted from Japanese individuals. We sought to replicate this association in two independent cohorts: one using whole blood from white UK-resident individuals, and one using a collection of human pancreatic islets, a more relevant tissue type to study with respect to the aetiology of diabetes.

**Methods:**

Levels of *CDKAL1-v1* were measured by real-time PCR using RNA extracted from human whole blood (*n* = 70) and human pancreatic islets (*n* = 48). Expression with respect to genotype was then determined.

**Results:**

In a simple linear regression model, expression of *CDKAL1-v1* was associated with the lead type 2 diabetes-associated SNP, rs7756992, in whole blood and islets. However, these associations were abolished or substantially reduced in multiple regression models taking into account rs9366357 genotype: a moderately linked SNP explaining a much larger amount of the variation in *CDKAL1-v1* levels, but not strongly associated with risk of type 2 diabetes.

**Conclusions/interpretation:**

Contrary to previous findings, we provide evidence against a role for dysregulated expression of *CDKAL1-v1* in mediating the association between intronic SNPs in *CDKAL1* and susceptibility to type 2 diabetes. The results of this study illustrate how caution should be exercised when inferring causality from an association between disease-risk genotype and non-coding RNA expression.

**Electronic supplementary material:**

The online version of this article (doi:10.1007/s00125-015-3508-9) contains peer-reviewed but unedited supplementary material, which is available to authorised users.

## Introduction

One of the most robust associations between common genetic variation and type 2 diabetes risk, reported in European and Asian populations, involves intronic single nucleotide polymorphisms (SNPs) in the *CDKAL1* gene, encoding CDK5 regulatory subunit associated protein 1-like 1 [1]. *CDKAL1* encodes a methylthiotransferase that catalyses the 2-methylthio (ms^2^) modification of various substrates, including the ms^2^ addition to *N* ^6^-threonyl-carbamoyladenosine at position 37 of tRNA^Lys^(UUU) [2]. The ms^2^ modification of tRNA^Lys^(UUU) stabilises the interaction with its cognate codons, allowing for efficient translation [3]. This is of particular relevance to the beta cell, where correct processing of proinsulin to insulin depends on a lysine residue located at the A-chain/C-peptide cleavage site [3]. Indeed *CDKAL1* risk allele carriers display an insulin secretory defect that is concomitant with higher levels of proinsulin [4], and beta cell-specific deletion of *Cdkal1* in mice results in glucose intolerance due to reduced insulin secretion and impaired proinsulin conversion [3]. These observations suggest that diabetes-associated risk alleles in humans are likely to reduce CDKAL1 activity.

It has been reported that the type 2 diabetes-associated risk alleles at this locus are associated with lower levels of a non-coding *CDKAL1* splice variant, *CDKAL1-v1*, which regulates CDKAL1 activity [5]. Zhou et al showed *CDKAL1-v1* contains binding sites for a microRNA, miR-494, that also targets the full-length *CDKAL1* transcript. By competing for miR-494, *CDKAL1-v1* regulates CDKAL1 activity such that if levels of *CDKAL1-v1* are lower, less miR-494 is sequestered away from *CDKAL1* mRNA and levels of CDKAL1 protein are reduced [5]. Whilst offering a plausible mechanism underlying the type 2 diabetes association, we sought to replicate their findings in another population and a more disease-relevant tissue type.

## Methods

### Participants/nucleic acid extraction

The study was carried out in accordance with the Declaration of Helsinki as revised in 2008. Clinical and genetic characteristics are presented in Electronic Supplementary Material (ESM) Table 1. RNA was extracted from whole blood of non-diabetic (all donor HbA_1c_ values <48 mmol/mol) white UK-resident donors using PAXgene Blood RNA Tubes (Qiagen, Venlo, the Netherlands) and PAXgene Blood miRNA Kit (Qiagen). DNA was extracted from EDTA tubes using the Wizard Genomic DNA Purification Kit (Promega, Madison, WI, USA). Snap-frozen pancreatic islets were supplied by ProCell Biotech (Newport Beach, CA, USA) and the National Institute of Diabetes and Digestive and Kidney Disease-funded Integrated Islet Distribution Program at City of Hope (Duarte, CA, USA). RNA was extracted using the mirVana miRNA Isolation Kit (Life Technologies, Carlsbad, CA, USA) and the small amounts of co-eluted genomic DNA whole genome amplified using the REPLI-g Mini Kit (Qiagen).

### Genotyping

SNPs were genotyped using TaqMan SNP Genotyping Assays (C_30175809_10, rs9366357; C_2504058_20, rs7756992) (Life Technologies) and TaqMan Genotyping Master Mix (Life Technologies).

### Quantitative RT-PCR

Total RNA was reverse transcribed using the SuperScript VILO Kit (Life Technologies). For real-time PCR, TaqMan Gene Expression Assays (ESM Table 2 presents assay IDs/sequences) and TaqMan Fast Advanced Master Mix (Life Technologies) were used. In islets and in whole blood from UK-resident donors, *CDKAL1-v1* expression was normalised using the geometric mean of five (*ACTB*, *B2M*, *GUSB*, *HMBS*, *RPL11*) and two (*18S*, *B2M*) housekeeping genes, respectively. Expression was calculated using the comparative C_t_ method [6] prior to log transformation to create parametric data suitable for regression analyses. For all regression analyses involving islet and UK blood samples, expression data were generated using the *CDKAL1-v1* assay without an oligonucleotide binding to a sequence overlapping rs9366357.

### Statistical analysis

Regression analyses were performed assuming an additive genetic model. In neither UK whole blood nor islet cohorts were age, sex, BMI or RNA integrity number values associated with *CDKAL1-v1* levels.

## Results

The TaqMan assay (Hs01557326) previously used to quantify *CDKAL1-v1* [5] includes an oligonucleotide that binds to a sequence containing the common SNP, rs9366357, which is in moderate linkage disequilibrium (LD) with lead type 2 diabetes-associated SNP, rs7756992 (1000 Genomes Pilot 1: *r* ^2^ = 0.3, JPT population; *r* ^2^ = 0.28, CEU population). We therefore wanted to determine whether the expression quantitative trait loci (eQTL) finding could be an artefact of allelic dropout and LD with rs7756992. To address this, we designed another assay, not including primers/probe overlapping rs9366357, to measure *CDKAL1-v1*. In 70 UK blood samples we found that levels of *CDKAL1-v1*, quantified using the two TaqMan assays, were very highly correlated (*r* ^2^ = 0.93). Hence, we do not believe that the previous results [5] are affected by allelic dropout.

**Fig. 1:**
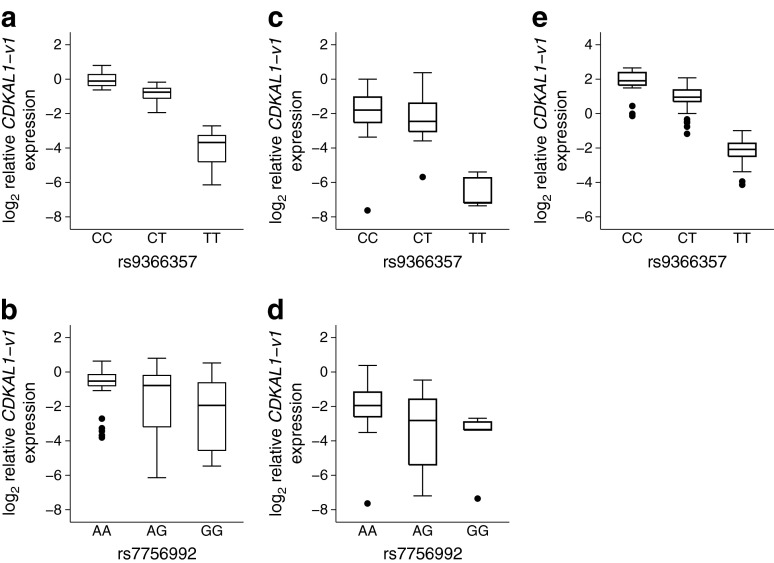
*CDKAL1-v1* levels stratified by genotype, (**a**, **b**) in whole blood from 70 white UK-resident donors, (**c**, **d**) in pancreatic islets from 48 white donors and (**e**) in whole blood from 103 Japanese donors. *y*-axis values were calculated using the comparative C_t_ method with values relative to the expression of *CDKAL1-v1* in one donor sample

Having eliminated the possibility that the results from the Japanese study were due to a technical artefact we sought to replicate their correlation between rs7756992 genotype and *CDKAL1-v1* levels. Given our sample size of 70, and based on the per-allele effect size observed in the Japanese study, we calculated we had >95% power to detect this association (with a type I error rate of 5%). Indeed, under a simple linear regression model we also found an effect for rs7756992 on *CDKAL1-v1* levels (*β* = −0.75, *p* = 0.005) (Fig. 1, Table 1), but no association with levels of *CDKAL1* mRNA (*β* = 0.04, *p* = 0.61). Furthermore, in contrast to the previous report [5], there was no correlation between levels of *CDKAL1* and *CDKAL1-v1* (*r* ^2^ = 0.00, *p* = 0.85).

**Table 1:** Results of linear regression analyses with *CDKAL1-v1* expression as the dependent variable and rs7756992 and rs9366357 as explanatory variables

Cohort	SNP	Simple linear regression		Multiple linear regression	
		*β* ^a^ ± SE	*p* value	*β* ^a^ ± SE	*p* value
Whole blood, white UK, *n* = 70	rs7756992	−0.75 ± 0.26	0.005	−0.34 ± 0.13	0.01
	rs9366357	−1.94 ± 0.13	3.2 × 10^−23^	−1.87 ± 0.13	1.3 × 10^−22^
Whole blood, Japan, *n* = 103	rs7756992	−1.04 ± 0.20	1.3 × 10^−6^	−0.07 ± 0.14	0.60
	rs9366357	−2.16 ± 0.13	9.3 × 10^−31^	−2.12 ± 0.15	1.9 × 10^−25^
Whole islets, white, *n* = 48	rs7756992	−1.07 ± 0.39	0.009	−0.61 ± 0.39	0.12
	rs9366357	−1.57 ± 0.39	2.4 × 10^−4^	−1.33 ± 0.42	0.003

^a^Effect size denotes per minor allele change in log_2_-transformed expression values

We remained intrigued by the presence of rs9366357 14 bp from the unique splice site of *CDKAL1-v1*. We used ESEfinder [7] to ascertain whether the alternative alleles of rs9366357 might affect the binding of any serine/arginine-rich (SR) proteins which may be important for regulating *CDKAL1-v1* levels. Indeed the T allele was predicted to abolish two strong binding sites for the SR protein, SR splicing factor 6. Given this finding, we determined whether there was any association between rs9366357 and levels of *CDKAL1-v1*. In the 70 UK blood samples we found an association between rs9366357 and *CDKAL1-v1* levels in simple linear regression (*β* = −1.94, *p* = 3.2 × 10^−23^) and in multiple linear regression, taking into account rs7756992 (*β* = −1.87, *p* = 1.3 × 10^−22^). The lead type 2 diabetes-associated SNP, rs7756992, was still associated with *CDKAL1-v1* levels when taking into account rs9366357, although the effect size was reduced and far smaller than the effect of rs9366357 (*β* = −0.34, *p* = 0.01) (Fig. 1, Table 1). Subsequently we genotyped rs9366357 in the 103 Japanese blood samples described in the first report detailing the *CDKAL1-v1*–rs7756992 association [5]. Again a striking effect of rs9366357 on *CDKAL1-v1* levels was seen (multiple linear regression *β* = −2.12, *p* = 1.9 × 10^−25^), but in this data set rs7756992 was no longer associated with *CDKAL1-v1* levels when taking into account rs9366357 (*β* = −0.07, *p* = 0.60) (Fig. 1, Table 1).

We next sought to determine whether *CDKAL1-v1* is similarly regulated in human pancreatic islets—the primary tissue of interest with respect to the type 2 diabetes association. In our cohort of 48 islets from white donors we found, in simple linear regression analyses, both rs9366357 (*β* = −1.57, *p* = 2.4 × 10^−4^) and rs7756992 (*β* = −1.07, *p* = 0.009) to be associated with *CDKAL1-v1* levels. However, in a multiple regression model only rs9366357 (*β* = −1.33, *p* = 0.003), and not rs7756992 (*β* = −0.61, *p* = 0.12), was associated with *CDKAL1-v1* expression (Fig. 1, Table 1).

Lastly, we investigated the association between rs9366357 and type 2 diabetes risk in a large case–control study. In data available from the DIAGRAM consortium, and involving 12,171 cases and 56,862 controls of North European descent, rs9366357 is only very weakly associated with risk of developing type 2 diabetes (OR 1.05, *p* = 0.003) compared with the lead SNP, rs7756992 (OR 1.20, *p* = 1.3 × 10^−22^). If differential *CDKAL1-v1* expression was heavily involved in mediating the association between variants at the *CDKAL1* locus and type 2 diabetes susceptibility, we would expect rs9366357, which explains a much larger amount of the variation in levels of *CDKAL1-v1* compared with rs7756992, to be more strongly associated with type 2 diabetes risk. The small effect of rs9366357 on diabetes risk leads us to conclude that dysregulated expression of *CDKAL1-v1* is unlikely to be the only genotype-dependent defect driving the association between genetic variation at the *CDKAL1* locus and type 2 diabetes risk.

## Discussion

Despite simple regression analyses replicating the association between lead genome-wide association study (GWAS) SNP and *CDKAL1-v1* levels, our more detailed investigations mean we can provide strong evidence against a causal role for this eQTL. Although eQTL studies provide an important tool for translating GWAS hits, inferring causal mechanisms should be made with caution, particularly with respect to *cis*-eQTLs involving non-coding RNAs. Probably due to lower functional constraints, *cis*-eQTL effect sizes are likely to be much larger for non-coding RNAs than for protein-coding transcripts; indeed, this has been reported when comparing *cis*-eQTLs involving large intergenic RNAs with *cis*-eQTLs involving protein-coding transcripts [8]. This has the potential, as clearly illustrated by our results, to make non-causal eQTLs appear causal, even if LD between the two SNPs is modest (*r* ^2^ < 0.7).

Given that highly cell type-specific regulation of gene expression is possible, our study may be confounded by the cellular heterogeneity of whole blood and whole islets. Whilst a study of *CDKAL1-v1* expression in sorted pancreatic beta cells would be ideal, we consider our analysis unlikely to be strongly confounded: human islets are ∼75% beta cells and 87% of the variance in beta cell gene expression can be explained by using islet expression as a proxy [9].

Future work should consider the existence of other genes at the *CDKAL1* locus when attempting to decipher causal mechanisms. Indeed, whilst physiological characterisation of the biochemical defect strongly implicates *CDKAL1* as the causative gene, it has been reported that the associated intronic region contains enhancer elements for the nearby transcription factor, SOX-4, which is important in pancreas development and mature beta cell function [10].

## Electronic supplementary material

Below is the link to the electronic supplementary material.

ESM Table 1 (PDF 85 kb)

ESM Table 2 (PDF 8 kb)

## Abbreviations

eQTL: Expression quantitative trait loci
GWAS: Genome-wide association studies
LD: Linkage disequilibrium
ms^2^: 2-Methylthio
SNP: Single nucleotide polymorphism
SR: Serine/arginine-rich
