# Supplementary material for: A cautionary tale: the non-causal association between type 2 diabetes risk SNP, rs7756992, and levels of non-coding RNA, CDKAL1-v1
Source: Diabetologia. 2015 Jan 30;58(4):745–8. doi: 10.1007/s00125-015-3508-9 (PMC4351432; doi:10.1007/s00125-015-3508-9)
Supplement: Supplementary file 1 — (PDF 85 kb) [file 125_2015_3508_MOESM1_ESM.pdf]

ESM Table 1. Clinical and genetic characteristics of cohorts

	Whole blood, white UK (n=70)	Whole islets, white (n=48)
Age (years)	56 (20-83)	46 (19-64)
Sex (m/f)	22/48	26/22
BMI	26 (20-40)	26 (21-40)
Fasting plasma glucose (mmol/l)[a]	4.9 (4.1-6.1)	N/A
HbA1c (mmol/mol)	36 (28-44)	N/A
rs9366357 (CC/CT/TT)[b]	24/27/19	25/18/5
rs7756992 (AA/AG/GG)[b]	29/26/15	16/27/5
RNA integrity number (RIN)[c]	8.4 (6.8-9.4)	7.8 (5.4-9.3)

All data presented as median (range). N/A; not available.
[a] Determined for 67 donors.
[b] No deviation ($p>0.05$) from Hardy-Weinberg equilibrium detected in either sample set.
[c] Determined for 69/70 blood samples and 47/48 islet samples.
