# Supplementary material for: A cautionary tale: the non-causal association between type 2 diabetes risk SNP, rs7756992, and levels of non-coding RNA, CDKAL1-v1
Source: Diabetologia. 2015 Jan 30;58(4):745–8. doi: 10.1007/s00125-015-3508-9 (PMC4351432; doi:10.1007/s00125-015-3508-9)
Supplement: Supplementary file 2 — (PDF 8 kb) [file 125_2015_3508_MOESM2_ESM.pdf]

ESM Table 2. TaqMan Gene Expression Assay IDs (or sequences for custom assay) used

**Gene**	**Assay ID**
ACTB	Hs01060665_g1
B2M	Hs00984230_m1
GUSB	Hs99999908_m1
HMBS	Hs00609297_m1
RPL11	Hs00831112_s1
18S	Hs99999901_s1
CDKAL1	Hs00214949_m1
CDKAL1-v1	Hs01557326_m1
CDKAL1-v1 (custom assay with oligos not binding to rs9366357)	F_ TGGAGAATATATGGCTGGACAGCTA<br>R_AGTAGGTCTTCTCCAGGAACTTTCT<br>Probe_ CACTCAACCTGAAATAACTGT
